# Standardized risk-stratified cardiac assessment and early posttransplant cardiovascular complications in kidney transplant recipients

**DOI:** 10.3389/fcvm.2024.1322176

**Published:** 2024-01-24

**Authors:** Silvie Rajnochova Bloudickova, Bronislav Janek, Karolina Machackova, Petra Hruba

**Affiliations:** ^1^ Department of Nephrology, Institute for Clinical and Experimental Medicine, Prague, Czech Republic; ^2^ Department of Cardiology, Institute for Clinical and Experimental Medicine, Prague, Czech Republic; ^3^ Transplant Laboratory, Institute for Clinical and Experimental Medicine, Prague, Czech Republic

**Keywords:** kidney, transplantation, cardiovascular disease, cardiovascular evaluation, cardiovascular complications, major adverse cardiac event, end-stage renal disease

## Abstract

**Introduction:**

Cardiovascular disease (CVD) is the leading cause of morbidity and mortality in kidney transplant recipient (KTR). There is a dearth of standardized guidelines on optimal cardiovascular evaluation of transplant candidates.

**Methods:**

This single-center cohort study aims to determine the effectiveness of our standardized risk-stratified pretransplant cardiovascular screening protocol, which includes coronary angiography (CAG), in identifying advanced CVD, the proper pretransplant management of which could lead to a reduction in the incidence of major cardiac events (MACE) in the early posttransplant period.

**Results:**

Out of the total 776 KTR transplanted between 2017 and 2019, CAG was performed on 541 patients (69.7%), of whom 22.4% were found to have obstructive coronary artery disease (CAD). Asymptomatic obstructive CAD was observed in 70.2% of cases. In 73.6% of cases, CAG findings resulted in myocardial revascularization. MACE occurred in 5.6% (*N* = 44) of the 23 KTR with pretransplant CVD and 21 without pretransplant CVD. KTR with posttransplant MACE occurrence had significantly worse kidney graft function at the first year posttransplant (*p* = 0.00048) and worse patient survival rates (*p* = 0.0063) during the 3-year follow-up period compared with KTR without MACE. After adjustment, the independent significant factors for MACE were arrhythmia (HR 2.511, *p* = 0.02, 95% CI 1.158–5.444), pretransplant history of acute myocardial infarction (HR 0.201, *p* = 0.046, 95% CI 0.042–0.970), and pretransplant myocardial revascularization (HR 0.225, *p* = 0.045, 95% CI 0.052–0.939).

**Conclusion:**

Asymptomatic CVD is largely prevalent in KTR. Posttransplant MACE has a negative effect on grafts and patient outcomes. Further research is needed to assess the benefits of pretransplant myocardial revascularization in asymptomatic kidney transplant candidates.

## 1 Introduction

Cardiovascular disease (CVD) is the leading cause of morbidity and mortality in both patients with advanced chronic kidney disease (CKD) and kidney transplant recipients (KTR). The prevalence in these patient populations is approximately 30 times higher compared with age-adjusted non-CKD populations (1, 2). Furthermore, an increased incidence of infections during the first year after transplantation contributes to a higher morbidity and mortality rate of KTR. The persistent inflammatory state associated with kidney transplantation may be aggravated by both endogenous and exogenous stimuli, leading to further activation of immune system which is a prerequisite for developing CVD (3). Prior to transplantation, the patients are already exposed to a uremia-associated chronic proinflammatory environment, which is characterized by elevated levels of proinflammatory cytokines (interleukin-6, IL-6, fibroblast growth factor-23, FGF-23), C-reactive protein (CRP), oxidative stress, endothelial dysfunction, and a calcium–phosphate metabolism disorder (4). This preexisting inflammatory state may be enhanced by posttransplant factors such as an inflammatory cytokine storm induced by donor brain death, ischemia-reperfusion injury, donor-specific antibodies associated with allograft rejection, cytomegalovirus infection stimulating innate immunity via interferon-stimulated genes, and calcineurin inhibitors (CNI) commonly used as concomitant immunosuppressive agents that promote endothelial activation, dysregulation of lipid and glucose metabolism, and hypertension (3, 5, 6). These pre- and posttransplant factors contribute to the acceleration of atherosclerosis and to an increased risk of cardiovascular events in KTR.

Recently, due to a marked improvement in patient survival, the criteria for accepting transplant candidates have been expanded, and the number of high-risk patients with CVD referred for transplantation has thus increased. Therefore, a complex pretransplant examination, especially of the cardiovascular system, has become ever more crucial for the proper assessment of the transplant candidates' suitability for transplantation and for the minimization of the incidence of posttransplant cardiovascular events that could negatively impact transplant outcomes. The data concerning pretransplant myocardial revascularization remain ambiguous due to a lack of clear evidence as to its beneficial impact on the posttransplant course of patients, particularly asymptomatic patients, as even controlled randomized studies in non-CKD populations did not provide any such evidence (7–9). Current guidelines recommend the performance of resting electrocardiography (ECG) and echocardiography (ECHO) in all renal transplant candidates. However, there were no definite guidelines on how to approach asymptomatic candidates or candidates with known CVD. For this reason, the scope of the cardiological examination was based on the risk stratification defined in the 2012 scientific statement by the American Heart Association/American College of Cardiology Foundation (AHA/ACC) that was written specifically for patients with ESRD being evaluated for kidney transplantation (10). These recommendations were based on published studies, surveys, and registry data and took into account the medical history, physical examination, cardiac conditions, and presence of risk factors. Risk factors such as age over 60 years, hypertension, dyslipidemia, smoking, diabetes mellitus, history of CVD, left ventricular hypertrophy, and dialysis therapy of more than a year are already present in most patients who are referred for kidney transplantation, and thus they can be stratified as “high-risk” patients (11). The recently published AHA scientific statement from 2022 provides clinicians additional precise guidance by specifically addressing the concerns of kidney transplant candidates (12).

In ESRD patients, clinically silent CVD is very common, and normal findings on the ECG and ECHO do not exclude serious coronary involvement. The majority of published studies recommend extended cardiovascular screening, including non-invasive cardiac stress tests (dobutamine stress echocardiography, myocardial perfusion scan) and coronary angiography (CAG), only in patients with multiple risk factors (13, 14). The situation in CKD patients is further complicated by the fact that there are significant differences in the sensitivity and specificity of cardiac stress tests ranging from 38% to 95% accuracy, despite the strong positive predictive value of up to 96% when detecting obstructive coronary artery disease (CAD) (15, 16). Besides ECG and ECHO, the gold standard for assessing the condition of the cardiovascular system is CAG, which represents the only method that allows for an objective assessment of the condition of patients’ coronary arteries regardless of distinct symptoms that are often absent in the majority of end-stage renal disease (ESRD) patients. An alternate modality to CAG that can be used for imaging of the coronary arteries is CT angiography (coronary computed tomography angiography, CCTA), especially in the patients in whom non-significant finding is expected (17).

In this study, we aim to evaluate whether our standardized risk-stratified pretransplant cardiovascular protocol that includes CAG screening in addition to ECG and ECHO may be useful in the detection of advanced cardiovascular disease, the proper pretransplant management of which could lead to a reduction in the incidence of major cardiac events (MACE) in the early posttransplant period.

## 2 Materials and methods

### 2.1 Study design

**Figure 1:**
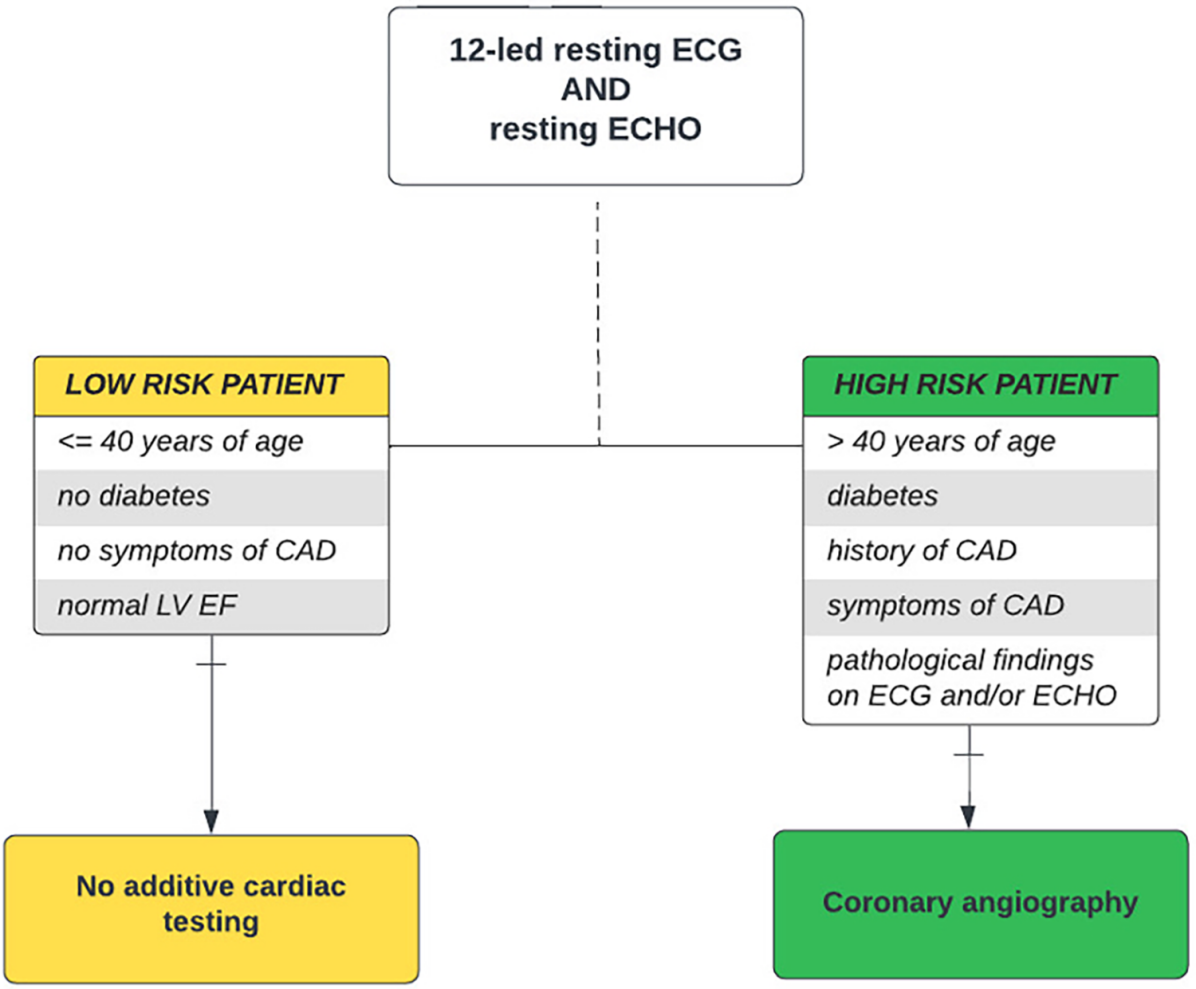
Pretransplant cardiovascular disease screening algorithm. Risk-stratified cardiovascular disease screening algorithm used in kidney transplant candidates at our center. It takes into account age, presence of diabetes, history and symptoms of cardiovascular disease, and pathological ECG and/or ECHO findings as risk factors, thereby distinguishing “low-risk” and “high-risk” kidney transplant candidates. CAD, coronary artery disease; DSE, dobutamine stress echocardiography; ECG, electrocardiography; ECHO, echocardiography; EF, ejection fraction; LV, left ventricle; MPS, myocardial perfusion scan.

This single-center, observational retrospective cohort study was conducted in adult patients who underwent kidney transplantation at our center between January 2017 and December 2019. Prior to transplantation, all individuals were evaluated using our standardized pretransplant risk-stratified cardiovascular protocol consisting of resting 12-lead electrocardiography (ECG), resting thoracic echocardiography, and coronary angiography. ECG and ECHO were performed in all kidney transplant candidates, while CAG was performed only in high-risk patients. A high-risk patient was defined as a patient with a presence of several risk factors: age over 40 years and/or with a history of diabetes, CVD or cardiovascular symptoms, and/or pathological findings on ECG and ECHO. A low-risk patient was defined as a patient aged 40 years and younger, with the absence of diabetes, with the absence of CVD or its symptoms, and with normal findings on resting ECG and ECHO (Figure 1). The pretransplant cardiovascular disease was recorded in patients with a history of myocardial infarction, heart failure or cardiac revascularization, percutaneous coronary intervention (PCI), and/or coronary artery bypass graft (CABG). A significant obstructive coronary artery disease was defined as stenosis of 50% or more of the left coronary artery (LCA) or 70% or more in at least one epicardial coronary artery or branch vessel detected using CAG (18, 19). Based on the findings, the patients were further indicated to stay on conservative therapy or to undergo myocardial revascularization, CABG, or PCI with (95.8% of patients) or without (4.2% of patients) last-generation drug-eluting stents (DES), according to the cardiological standard of care.

The primary endpoint was to determine the effect of our cardiovascular disease screening algorithm on the detection rate of obstructive CAD and on assessing the need for myocardial revascularization prior to transplantation. The secondary endpoint was to evaluate the impact of pretransplant CVD detection and management on the incidence of MACE in the early posttransplant period and to specify the prognostic indicators of MACE. MACE was defined as the need for a revascularization procedure (PCI, CABG), symptomatic arrhythmia (atrial fibrillation/flutter) with the need for intervention (electrocardioversion, radiofrequency ablation), myocardial infarction, heart failure, and sudden death (20, 21).

### 2.2 Statistical analysis

Continuous variables are expressed as medians (min, max) and compared using the Wilcoxon test, and categorical variables are expressed as *N* and a percentage of the total and compared using Pearson's chi-squared test. Survival analysis was performed using the Kaplan–Meier method, and the differences between groups were compared using the log-rank test. Univariable and multivariable Cox regression models were used to identify the risk and prognostic factors associated with posttransplant MACE. A *p*-value of less than 0.05 was considered statistically significant. The statistical analysis was performed using IBM SPSS Statistics, version 24 (International Business Machines Corp.) and RStudio software, version 4.1.3 (2022-03-10), development for R (RStudio, Inc., Boston, MA).

## 3 Results

### 3.1 Study cohort

A total of 776 kidney transplant recipients (KTR) enrolled in this study were followed for outcome measures for an average of 3 years posttransplant. The patients were analyzed based on the presence of pretransplant cardiovascular disease and posttransplant outcome measures.

The majority (93.6%) of KTR was transplanted from deceased donors, and 95.1% of KTR were on dialysis therapy (80% on hemodialysis, 20% on peritoneal dialysis). In our cohort, 94.7% of KTR treated with hemodialysis prior to transplantation were dialyzed using AV fistula, and 5.3% used a central venous catheter. The average vintage of dialysis before kidney transplantation was 2.2 years (median 2 years). After kidney transplantation, all KTR received our standard triple immunosuppressive therapy consisting of calcineurin inhibitor (CNI), purine synthesis inhibitor (mycophenolate mofetil, MMF), and steroids.

According to our risk-stratified cardiovascular algorithm, CAG was performed on a total of 541 out of 776 patients (69.7%). The obstructive CAD was detected in 121 of 541 KTR (22.4%). In 85 of 121 KTR (70.2%), CAD was fully asymptomatic and detected using our pretransplant screening protocol. The most commonly affected arteries were the left coronary artery and interventricular branch (LCA/RIA) (*N* = 78, 65.3%), right coronary artery (RCA) (*N* = 45, 37.2%), diagonal branch (*N* = 33, 27.3%), and obtuse marginal (OM) (*N* = 32, 26.4%) branch. Out of the total number of patients, 26 (21.5%) had two-vessel disease (2-VD), and 15 (12.4%) had three-vessel disease (3-VD), resulting in a total of 41 (33.9%) patients with multivessel disease (Table 1).

**Table 1:** Characteristics of 121 patients with pretransplant cardiovascular disease.

	Number of patients (*N*)	Percentage (%)	[min, max]
Male	99	81.8	
Age at transplantation median	68		48.76
Dialysis vintage	115	95	
Diuresis < 500 ml	65	53.7	
History of arrhythmia	13	10.7	
History of diabetes mellitus	54	44.6	
Asymptomatic CAD	85	70.2	
Myocardial infarction	26	21.5	
Myocardial revascularization (PCI/CABG)	11	9.1	
Conservative management of CAD	32	26.4	
Dual antiplatelet therapy prior transplantation	95	78.5	
LVEF < 60%	24	19.8	
Pulmonary hypertension	10	8.3	
Valvular disease	18	14.9	
Myocardial kinetics disorder	26	21.5	
2-VD ( > 50% artery stenosis of two of LCA/RCx/RIA/RCA)	26	21.5	
3-VD ( > 50% artery stenosis of three of LCA/RCx/RIA/RCA)	15	12.4	
Presence of 2-VD or 3-VD	41	33.9	
Posttransplant MACE	23	19	

CAD, coronary artery disease; LV EF, left ventricular ejection fraction; PCI, percutaneous coronary intervention; CABG, coronary artery bypass graft; 2-VD, 2-vessel disease; 3-VD, 3-vessel disease; RCx, ramus circumflexus; LCA, left coronary artery; RIA, ramus interventricularis anterior; RCA, right coronary artery; MACE, major adverse cardiac event.

**Figure 2:**
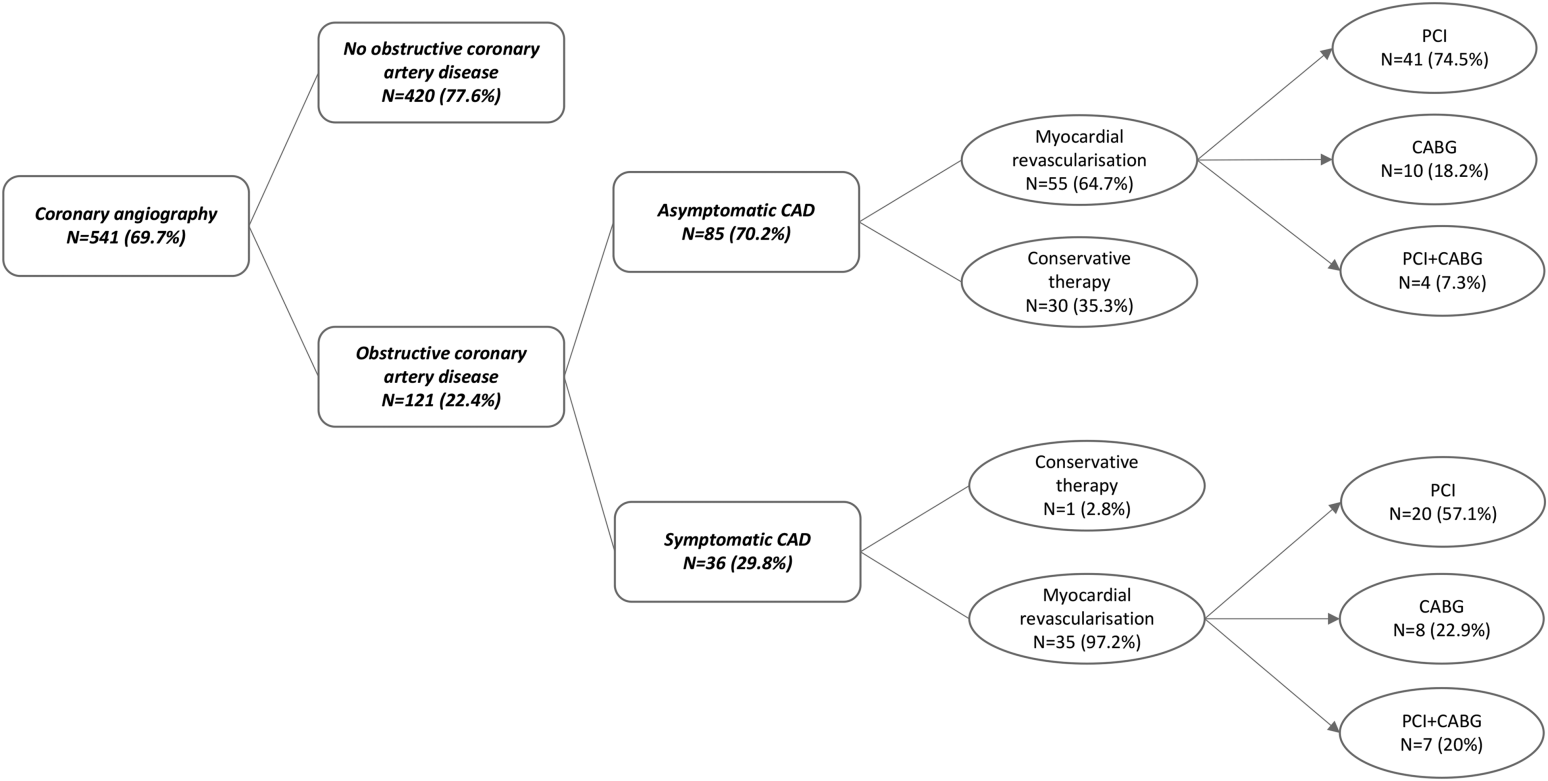
Pretransplant coronary angiography outcome in “high-risk” kidney transplant recipients. Obstructive coronary artery disease was detected in 22.4% of KTR who underwent coronary angiography according to the protocol. KTR with symptomatic CAD (29.8%) were in 97.2% managed with myocardial revascularization prior to transplantation. Asymptomatic CAD was detected in 70.2% of KTR, of which 64.7% were managed using myocardial revascularization as well. CAD, coronary artery disease; PCI, percutaneous coronary intervention; CABG, coronary artery bypass graft.

Based on CAG findings, myocardial revascularization was performed in 90 out of 121 patients (74.3%). The majority underwent PCI (*N* = 61, 67.8%), CABG was performed in 18 patients (20%), and 11 patients (12%) had a history of both PCI and CABG. Conservative therapeutic approach was opted for in 31 cases (25.6%). Asymptomatic obstructive CAD was treated conservatively in 30 patients (35.3%), and 55 patients (64.7%) received treatment either with PCI (*N* = 41, 74.5%), CABG (*N* = 10, 18.2%), or with both PCI and CABG (*N* = 4, 7.3%). Only one patient (2.8%) with a known pretransplant CAD (*N* = 36) was treated conservatively, whereas 35 (97.2%) patients were treated either with PCI (*N* = 20, 57.1%), CABG (*N* = 8, 22.9%), or with both PCI and CABG (*N* = 7, 20%). Prior to transplantation, 52 patients (43%) were treated with dual antiplatelet therapy (DAPT) due to the performance of myocardial revascularization (Figure 2).

**Figure 3:**
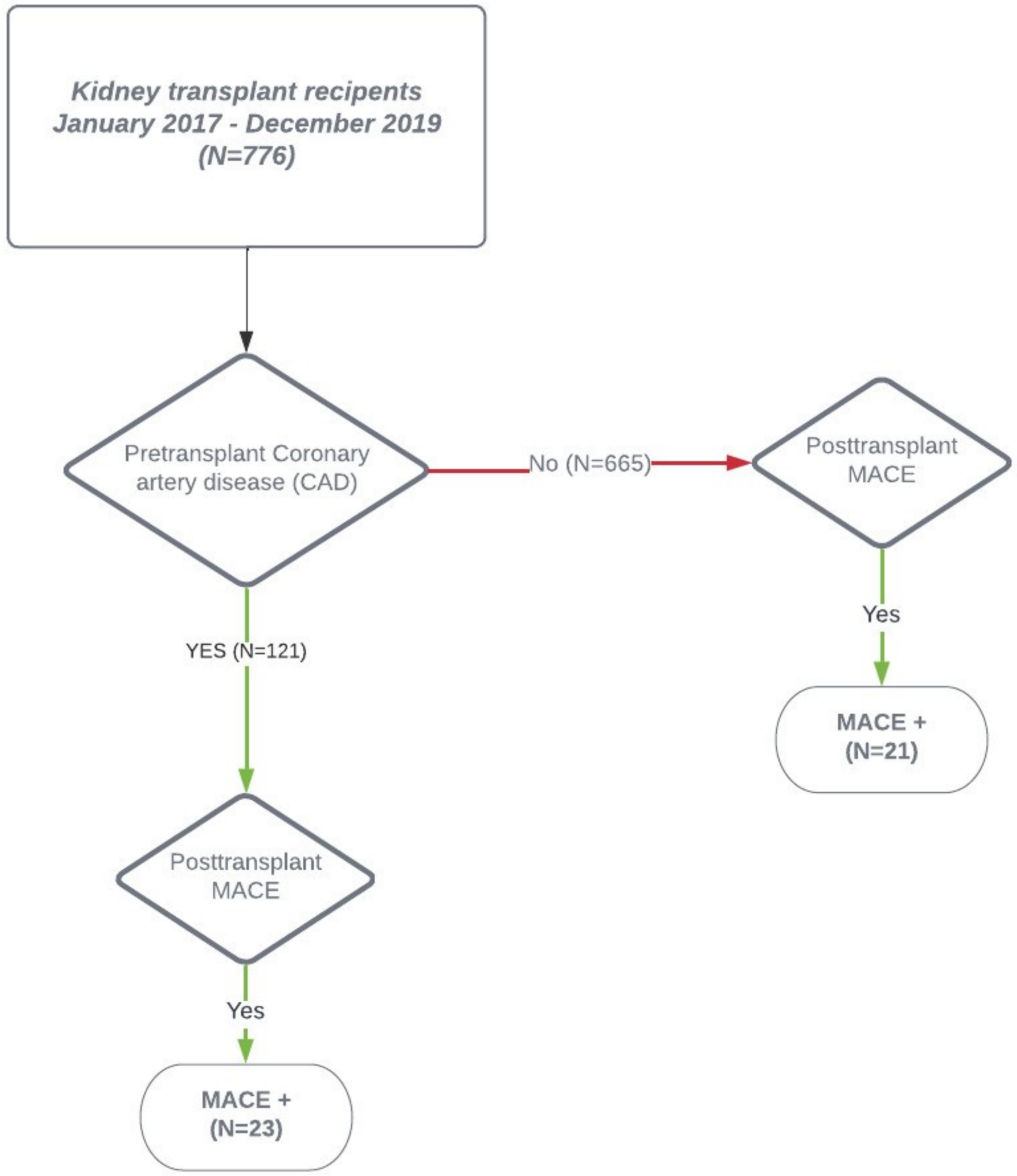
Post-transplant MACE manifestation. The 2-year posttransplant occurrence of MACE in the cohort of kidney transplant recipients. The overall MACE rate was low (5.7%) with a similar distribution between KTR with pretransplant obstructive coronary artery disease and KTR without pretransplant obstructive coronary artery disease. CAD, coronary artery disease; KTR, kidney transplant recipient; MACE, major adverse cardiac event.

**Figure 4:**
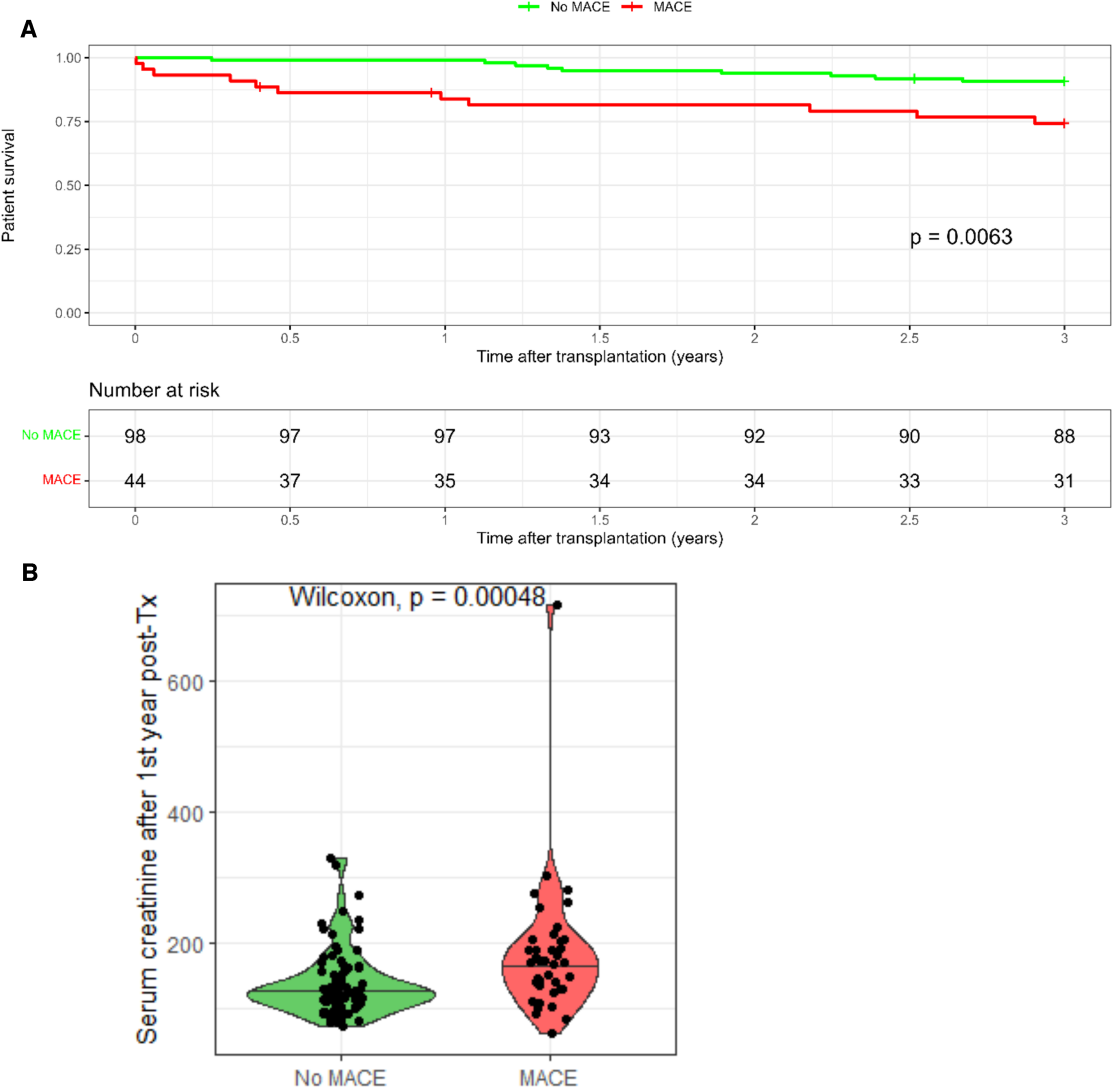
Outcomes of kidney transplant recipients with post-transplant MACE. (**A**) Kidney transplant recipients without MACE had significantly better survival compared to kidney transplant recipients experiencing MACE. (**B**) Kidney transplant recipients with MACE had significantly worse graft survival at 1st year post-transplant.

Out of the 776 KTR, MACE occurred in 44 (5.6%) patients only, 23 with pretransplant CVD and 21 without pretransplant CVD (Figure 3). Interestingly, KTR with pretransplant CVD and posttransplant MACE did not significantly differ in the extent of coronary artery involvement (2-VD and 3-VD) compared with KTR with pretransplant CVD but without posttransplant MACE. Comparing KTR with MACE occurrence, KTR with no pretransplant CVD were younger (*p* = 0.008), had preserved residual diuresis (*p* = 0.04), preserved left ventricular ejection fraction (*p* = 0.048), and a tendency towards more frequent history of arrhythmia (*p* = 0.052). The most fundamental difference was that KTR with posttransplant MACE occurrence had significantly worse survival rates (log-rank *p* = 0.0063) during the 3-year follow-up period compared with KTR without MACE occurrence (Figure 4A) and also had worse kidney graft function at the first year posttransplant (*p* = 0.00048, Figure 4B).

### 3.2 Analysis of risk factors for MACE

The univariable Cox regression model identified the most significant variables positively affecting MACE, including pretransplant CVD (HR 0.070, *p* < 0.001, 95% CI 0.035–0.136), asymptomatic CVD detected by pretransplant evaluation (HR 0.343, *p* = 0.001, 95% CI 0.035–0.136), pretransplant myocardial revascularization using PCI/CABG (HR 0.251, *p* < 0.001, 95% CI 0.135–0.470) with dual antiplatelet therapy (HR 0.397, *p* = 0.014, 95% CI 0.190–0.828), and, surprisingly, the history of myocardial infarction (HR 0.181, *p* = 0.018, 95% CI 0.044–0.750). History of arrhythmia (HR 3.051, *p* = 0.001, 95% CI 1.613–5.770) and radiofrequency ablation (HR 6.449, *p* < 0.001, 95% CI 2.493–16.680) were found to negatively affect MACE occurrence. Pulmonary hypertension showed some tendency, but the findings did not reach statistical significance (HR 1.938, *p* = 0.091, 95% CI 0.900–4.172) (Table 2).

**Table 2:** Univariable analysis of risk factors for MACE.

Variable	HR	95% CI	*p*-value
Age at transplantation, years	0.990	0.960–1.021	0.523
Donor age, years	0.998	0.979–1.018	0.872
Dialysis vintage, years	1.061	0.937–1.201	0.351
Pretransplant diabetes	0.961	0.911–1.014	0.145
Pretransplant diabetes on insulin therapy	0.426	0.168–1.089	0.075
Pretransplant CVD	0.070	0.035–0.136	<0.001
CVD detection within pretransplant evaluation	0.343	0.187–0.631	0.001
Pretransplant myocardial revascularization (PCI/CABG)	0.251	0.135–0.470	<0.001
Dual antiplatelet therapy prior to transplantation	0.397	0.190–0.828	0.014
Pretransplant arrhythmia	3.051	1.613–5.770	0.001
Pretransplant RF ablation	6.449	2.493–16.680	<0.001
History of myocardial infarction	0.181	0.044–0.750	0.018
Pulmonary hypertension	1.938	0.900–4.172	0.091
Myocardial kinetics disorder	0.753	0.336–1.689	0.491

CVD, cardiovascular disease; PCI, percutaneous coronary intervention; CABG, coronary artery bypass graft; RF, radiofrequency; HR, hazard ratio; CI, confidence interval.

The multivariable Cox regression model was constructed based on the results from the univariable regression model. After adjustments for radiofrequency ablation, pulmonary hypertension, and pretransplant antiplatelet therapy, the independent significant factors for MACE remained arrhythmia (HR 2.511, *p* = 0.02, 95% CI 1.158–5.444), pretransplant history of acute myocardial infarction (HR 0.201, *p* = 0.046, 95% CI 0.042–0.970), and pretransplant myocardial revascularization (HR 0.225, *p* = 0.045, 95% CI 0.052–0.939) (Table 3).

**Table 3:** Multivariable analysis of risk factors for MACE.

Variable	HR	95% CI	*p*-value
Pretransplant arrhythmia	2.511	1.158–5.444	0.020
Pretransplant radiofrequency ablation	1.565	0.446–5.498	0.485
History of myocardial infarction	0.201	0.042–0.970	0.046
Pretransplant myocardial revascularization (PCI/CABG)	0.225	0.052–0.939	0.045
Dual antiplatelet therapy prior to transplantation	0.358	0.104–1.233	0.103
Pulmonary hypertension	1.227	0.509–2.960	0.649

MACE, major adverse cardiac event; PCI, percutaneous coronary intervention; CABG, coronary artery bypass graft; HR, hazard ratio; CI, confidence interval.

## 4 Discussion

The increasing numbers of high-risk cardiac patients are being considered as potential candidates for kidney transplantation. The main role of pretransplant evaluation is to determine whether the benefits of transplantation outweigh the risks of posttransplant cardiovascular complications in particular. Thus, screening for cardiovascular disease is essential for kidney transplantation acceptance. Pretransplant cardiovascular assessment approaches differ across transplantation centers due to the lack of standardized guidelines, which are currently based rather on recommendations that prioritize local practice (14, 22, 23).

Our study analyzed the effectiveness of our pretransplant risk-stratified protocol using screening coronary angiography in detecting significant cardiovascular disease in patients undergoing renal transplants and the impact of this approach on the incidence of posttransplant cardiac events. We believed that CAG is the most effective approach for CAD detection because in ESRD patients, the sensitivity and specificity of stress tests used for the detection of significant CAD are insufficient despite the high negative predictive value (15, 24, 25).

Based on our protocol, obstructive CAD was determined in 22.4% KTR who underwent CAG as “high-risk” patients, out of which 70.2% were clinically asymptomatic. The majority of patients with significant CAD (74.3%) were further treated with myocardial revascularization, PCI in 67.8%, CABG in 20%, and a combination of both PCI and CABG in 12%. Our findings are consistent with the knowledge of the high prevalence of CAD in patients with ESRD, particularly in those on dialysis (26, 27). Low occurrence of cardiac symptoms in dialyzed patients, including in those with advanced obstructive CAD, might be the cause for the underestimation of cardiovascular disease in this patient population. However, there still remains hesitation concerning the routine use of pretransplant coronary angiography for the detection of CAD in transplant candidates. This is because recent studies have not found conclusive evidence regarding the long-term impacts of prophylactic revascularization on patient morbidity and mortality (8, 18, 28). The prospective randomized ISCHEMIA-CKD trial including 777 patients with advanced chronic kidney disease (eGFR < 30 ml/min or dialysis dependence) did not conclude any cardioprotective benefits of myocardial revascularization in comparison with conservative strategies referencing the 3-year event rate of non-fatal myocardial infarction or death being 29% and 30%, respectively (29). However, several other studies detailed more frequent and more severe coronary adverse events and higher rates of death at 5 years posttransplant in patients in whom advanced CAD was being managed medically compared with those who had myocardial revascularization prior to transplantation (30–32).

On the other hand, there is some awareness regarding the association between CAG, myocardial revascularization, and a threefold increase in periprocedural morbidity and mortality in ESRD patients compared with non-CKD patients (33). In our cohort, we have not registered any major periprocedural complications, probably due to the elective nature of the conducted CAG. In general, the risk of experiencing major periprocedural complications appears to be low, varying between 0.1% and 0.25%, respectively, being 0.05% in diagnostically performed CAG (34). Similarly, another argument for not performing CAG routinely might be the risk of deterioration of residual renal function (RRF). RRF is an important predictor of survival in dialyzed patients; therefore, it is an effort to preserve RRF as long as possible. Recent studies analyzing the effect of contrast media on RRF have concluded that RRF is not significantly influenced by intravascular administered iso-osmolar contrast media with adequate prehydration in ESRD patients (35–38).

Nevertheless, a significant decrease in the risk of myocardial infarction and death in ESRD patients with multivessel CAD treated with CABG compared with PCI has been well determined. The use of multiple PCI procedures has shown similar benefits in patients with multivessel CAD (39, 40). Observational accounts also point to the long-term benefits of surgical revascularization in ESRD patients in cases of obstructive CAD compared with conservative management (31, 41, 42). Other concerns include longer waiting time of transplant candidates caused by the administration of dual antiplatelet therapy due to myocardial revascularization. However, we have observed that the pretransplant administration of dual antiplatelet therapy has a significantly beneficial impact on posttransplant occurrence of cardiac events, similar to the impact of myocardial revascularization performed in cases of significant CAD (20, 43). Moreover, the use of last-generation drug-eluting stents reduced the need for DAPT therapy to only 3–6 months.

Major cardiac adverse events were observed in only 5.6% (44) of all KTR, out of which 23 had pretransplant CVD and 21 had no pretransplant CVD. The groups presented similar types of MACE and posttransplant survival rates (Table 4). This observation may be explained by preserved echocardiographic prognostic factors such as left ventricular geometry and ventricular kinetics (7, 44, 45). We believe that the low number of posttransplant MACE in our cohort is just due to the detection and adequate treatment of cardiovascular findings prior to transplantation.

**Table 4:** Characteristics of 44 patients experiencing MACE.

	Pretransplant CVD (*N* = 23)	No pretransplant CVD (*N* = 21)	*p*-value
Male *N* (%)	18 (78.3)	13 (61.9)	0.325
Age at transplantation median [min, max]	67 [49,73]	65 [49,74]	0.008
Dialysis vintage < 1 year *N* (%)	2 (8.7)	1 (4.8)	1.000
Dialysis vintage 1–3 years *N* (%)	11 (47.8)	10 (47.6)	1.000
Dialysis vintage > 3 years *N* (%)	8 (34.8)	10 (47.6)	0.541
Diuresis < 500 ml *N* (%)	9 (39.1)	6 (28.6)	0.040
History of arrhythmia *N* (%)	4 (17.4)	10 (47.6)	0.052
History of diabetes	11 (47.8)	8 (38.1)	0.557
Pretransplant echocardiography			
LVEF > 60% median [min, max]	18 (78.3)	20 (95.2)	0.048
Pulmonary hypertension *N* (%)	3 (13)	5 (23.8)	0.448
Significant valvular disease *N* (%)	6 (26.1)	4 (19)	0.724
Myocardial kinetics disorder *N* (%)	6 (26.1)	0 (0)	0.097
MACE			
Arrhythmia *N* (%)	11 (47.8)	13 (61.9)	0.382
Acute myocardial infarction *N* (%)	6 (26.1)	1 (4.8)	0.097
Heart failure *N* (%)	5 (21.7)	4 (19)	1.000
Cardiovascular death *N* (%)	1 (4.3)	2 (9.5)	0.599
Serum creatinine after MACE median [min, max]	174 [63,717]	169 [86,278]	0.583
Patients’ outcome after total posttransplant follow-up			
Alive *N* (%)	21 (91.3)	19 (90.5)	0.169
Total death due to non-CVD cause *N* (%)	2 (8.7)	0 (0)	0.489
Total death due to CVD cause N (%)	6 (26.1)	4 (19)	0.724

MACE, major adverse cardiac event; CVD, cardiovascular disease; LV EF, left ventricular ejection fraction.

Regarding the independent risk factors for posttransplant MACE occurrence in our cohort, we observed that arrhythmias and radiofrequency ablation performed prior to transplantation were found to significantly increase the risk of MACE (Table 2). Due to the high prevalence of ECG abnormalities in ESRD patients, we included only KTR with a documented history of persistent atrial fibrillation or pretransplant atrial fibrillation treated with radiofrequency ablation. This observation has an important clinical impact as the presence of atrial fibrillation at the time of transplantation not only increases the risk of cardiac complications, but also increases the risk of death at 5 years posttransplant (46, 47). Furthermore, the presence of cardiovascular disease and a history of myocardial infarction were identified as the strongest factors in preventing the occurrence of posttransplant MACE (Table 3). Based on our findings, it may be suggested that optimal myocardial revascularization and favorable echocardiographic findings made the acceptance of candidates for renal transplantation possible. For this reason, it is crucial to evaluate cardiological findings under conditions of effective dialysis and optimal hydration to avoid misinterpretation. There is evidence that dialysis efficiency, not dialysis modality (hemodialysis or peritoneal dialysis), is associated with the incidence of CVD (48, 49, 50). Heart failure is an important predictor of mortality in dialyzed and transplanted patients. Approximately 80% of patients with heart failure and systolic–diastolic dysfunction die within 3 years (51). Despite the clearly positive effect of a functional transplanted kidney on cardiac function, patients with a history of heart failure have a more than two times higher risk of heart failure or death, even 5 years after transplantation (52, 53). This risk increases as the ejection fraction decreases (54). Heart failure was observed as one of the most frequent MACE in our cohort. There was no significant difference observed between the groups in terms of the incidence of MACE or death caused by heart failure (50%), reaching approximately 20% of KTR experiencing these outcomes. Our findings appear to be in accordance with published data.

Pulmonary hypertension, with a prevalence rate ranging from 18%–56% in ESRD patients, is known to be a strong independent prognostic factor of morbidity and mortality in both patients with CKD and KTR, as well as of lower graft survival (55–57). In our cohort, pulmonary hypertension was found in 8.3% (10) of patients with CVD, out of which only three (13%) patients developed MACE. Approximately 24% of patients without pretransplant CVD but with posttransplant MACE occurrence had pulmonary hypertension. The higher prevalence of pulmonary hypertension might be considered a prognostic factor for MACE in patients without pretransplant CVD, despite preserved myocardial kinetics (21, 58). However, the rate of pulmonary hypertension did not reach statistical significance, probably due to the small number of patients whose endpoint was MACE occurrence (Table 2). The similar percentage rate of patients who experienced the occurrence of MACE irrespective of CVD in our cohort supports the finding that atherosclerotic CAD represents only a portion of cardiovascular complications occurring in KTR. Dysrhythmias with high prevalence of systolic or diastolic dysfunction, left ventricular hypertrophy, and electrical instability are associated with approximately 50% of cardiovascular deaths in KTR (59). Siddiqui et al. (60) recently published a meta-analysis evaluating eight studies pertaining to the subject of strategy in kidney transplant candidates with established CAD. Independent of whether the management of CAD was invasive or conservative, they found no differences regarding all-cause mortality, cardiovascular mortality, and the occurrence of MACE, including myocardial infarction, heart failure, and arrhythmias. Based on this analysis, their recommendation is to perform revascularization procedures exclusively on patients with anatomically high-risk CAD in whom the intervention might be beneficial for the improvement of survival, but to not revascularize asymptomatic CAD patients routinely if the sole aim is to reduce the occurrence of perioperative cardiac events.

Among the factors that have an impact on posttransplant cardiovascular complications, the influence of concomitant immunosuppression cannot be neglected. Currently, KTR are standardly treated with a triple immunosuppressive regimen consisting of calcineurin inhibitor, purine synthesis inhibitor (MMF), and steroids. There are multiple studies suggesting the effects of CNI on human hearts, particularly on hypertrophy or increased left ventricle mass (61, 62). Recently published review dealing with cardiovascular effect of immunosuppressives reported that the increase of left ventricle mass may be primarily driven by CNI-induced fibrosis and collagen deposition rather than cardiomyocyte remodeling. On the other hand, there are no data suggesting the link between purine synthesis inhibitors and cardiac hypertrophy or fibrosis (63). This potential impact of CNI on the progression of CVD should be taken into account as a part of pretransplant decision-making process, particularly in marginal kidney transplant candidates.

In our study, we observed negative impacts of posttransplant cardiac events in all patients in whom MACE occurred, irrespective of the presence of CVD. Despite the similar characteristics of the patients with pretransplant CVD, those who experienced the occurrence of MACE had significantly worse renal graft function at 1 year and higher mortality rates. The patients without pretransplant CVD but with posttransplant MACE occurrence showed unfavorable outcomes comparable with those of the patients with pretransplant CVD and posttransplant MACE occurrence (Table 4). The patients with posttransplant MACE showed significantly worse renal graft function and patient survival rates in comparison with those without cardiac complications (Figure 4). Due to the lack of prospective randomized trials in renal transplant candidates, the optimal modality for the screening and management of ischemic heart disease in this patient population remains a matter of debate, and current practice guidelines suggest excluding asymptomatic CVD patients from routine invasive testing and proceeding them to transplantation (64). The 2022 AHA scientific statement recommends performing cardiac catheterization in asymptomatic kidney transplant candidates without a history of CVD individually based on the findings of the resting ECHO examination. Regarding the kidney transplant candidates with known CVD, it is recommended to have direct cardiac catheterization in patients with cardiac symptoms or in cases of pathological findings on a stress test in patients who have no cardiac symptoms. Currently, there is no established practice of routinely performing revascularization procedures on stable and asymptomatic kidney transplant candidates only for the purpose of reducing long-term cardiovascular mortality. However, pretransplant revascularization should be individualized depending on the risk associated with delayed transplantation and the benefits of reducing cardiovascular risk (12). Currently, there is a lack of guidelines or recommendations addressing the possible impact of pretransplant cardiovascular revascularization on short- or medium-term cardiovascular mortality.

We believe our observations might prove useful for optimizing the evaluation approaches used to assess pretransplant cardiovascular patients in kidney transplantation prior to listing candidates for transplantation, including candidates with asymptomatic advanced CVD.

## 5 Conclusion

Advanced cardiovascular disease is prevalent and largely asymptomatic in patients undergoing kidney transplantation. Posttransplant cardiovascular events are associated with decreased graft survival rates and adverse patient outcomes. Further studies are required to assess the benefits of pretransplant myocardial revascularization in asymptomatic kidney transplant candidates.

### 5.1 Strengths and limitations

This study aimed to describe our single-center experience with an algorithm that was developed as a part of a collaboration between transplant nephrologists and cardiologists to assess cardiovascular risk prior to kidney transplantation. The strengths of our study include the number of patients in whom CAG was performed in accordance with the pretransplant protocol and the availability of all data obtained from both ECHO and CAG procedures.

The presented study was conducted retrospectively at a single center. Another limitation of the study is its short-term design, allowing us to present only short-term patient outcomes. Thus, we are not yet able to provide insights on the long-term impacts of our pretransplant cardiovascular screening algorithm on patient morbidity and mortality rates. We specifically focused our analysis on patients who underwent kidney transplantation, excluding those who were not accepted for the procedure.

## Data Availability

The original contributions presented in the study are included in the article/Supplementary Material, further inquiries can be directed to the corresponding author.

## References

[1] Collins AJ Li S Ma JZ Herzog C. Cardiovascular disease in end stage renal disease patients. Am J Kidney Dis. (2001) 38:S26–9. 10.1053/ajkd.2001.27392 11576917

[2] Go AS Chertow GM Fan D McCulloch CE Hsu CY. Chronic kidney disease and the risk of death, cardiovascular events, and hospitalization. N Engl J Med. (2004) 351:1296–305. 10.1056/NEJMoa041031 15385656

[3] Ponticelli C Campise MR. The inflammatory state is a risk factor for cardiovascular disease and graft fibrosis in kidney transplantation. Kidney Int. (2021) 100(3):536–45. 10.1016/j.kint.2021.04.016 33932457

[4] Jankowski J Floege J Fliser D Böhm M Marx N. Cardiovascular disease in chronic kidney disease. Circulation. (2021) 143(11):1157–72. 10.1161/CIRCULATIONAHA.120.050686 33720773 PMC7969169

[5] Loupy A Vernerey D Viglietti D Aubert O Van Huyen JPD Empana JP Determinants and outcomes of accelerated arteriosclerosis: major impact of circulating antibodies. Circ Res*.* 2015;117(5):470–82. 10.1161/CIRCRESAHA.117.306340 26056252

[6] Rodrigues-Diez R González-Guerrero C Ocaña-Salceda C Rodrigues-Diez RR Egido J Ortiz A. Calcineurin inhibitors cyclosporine A and tacrolimus induce vascular inflammation and endothelial activation through TLR4 signaling. Sci Rep. (2016) 6:27915. 10.1038/srep27915 27295076 PMC4904742

[7] Hage FG Smalheiser S Zoghbi GJ Perry GJ Deierhoi M Warnock D Predictors of survival in patients with end-stage renal disease evaluated for kidney transplantation. Am J Cardiol. (2007) 100(6):1020–5. 10.1016/j.amjcard.2007.04.045 17826390

[8] Aalten J Peeters SA van der Vlugt MJ Hoitsma AJ. Is standardized cardiac assessment of asymptomatic high-risk renal transplant candidates beneficial? Nephrol Dial Transplant. (2011) 26:3006–12. 10.1093/ndt/gfq822 21321004

[9] Boden WE O’Rourke RA Teo KK Hartigan PM Maron DJ Kostuk WJ Optimal medical therapy with or without PCI for stable coronary disease. N Engl J Med. (2007) 356(15):1503–16. 10.1056/NEJMoa070829 17387127

[10] Lentine KL Costa SP Weir MR Robb JF Fleisher LA Kasiske BL Cardiac disease evaluation and management among kidney and liver transplantation candidates: a scientific statement from the American Heart Association and the American College of Cardiology Foundation: endorsed by the American Society of Transplant Surgeons, American Society of Transplantation, and National Kidney Foundation. Circulation. (2012) 126(5):617–63. 10.1161/CIR.0b013e31823eb07a 22753303

[11] Neale J Smith AC. Cardiovascular risk factors following renal transplant. World J Transplant. (2015) 5(4):183–95. 10.5500/wjt.v5.i4.183 26722646 PMC4689929

[12] Cheng XS Van Wagner LB Costa SP Axelrod DA Bangalore S Norman SP Emerging evidence on coronary heart disease screening in kidney and liver transplantation candidates: a scientific statement from the American Heart Association: endorsed by the American Society of Transplantation. Circulation. (2022) 146(21):e299–324. 10.1161/CIR.0000000000001104 36252095 PMC10124159

[13] Rangaswami J Mathew RO Parasuraman R Tantisattamo E Lubetzky M Rao S Cardiovascular disease in the kidney transplant recipient: epidemiology, diagnosis and management strategies. Nephrol Dial Transplant. (2019) 34:760–73. 10.1093/ndt/gfz053 30984976

[14] Chadban SJ Ahn C Axelrod DA Foster BJ Kasiske BL Kher V KDIGO on the evaluation and management of candidates for kidney transplantation. Transplantation. (2020) 104:708–14. 10.1097/TP.0000000000003137 32224812 PMC7147399

[15] Kanigicherla DAK Bhogal T Stocking K Chinnadurai R Gray S Javed S Non-invasive cardiac stress studies may not offer significant benefit in pre-kidney transplant evaluation: a retrospective cohort study. PLoS One. (2020) 15(10):e0240912. 10.1371/journal.pone.0240912 33113550 PMC7592791

[16] Dilsizian V Gewirtz H Marwick TH Kwong RY Raggi P Al-Mallah MH Cardiac imaging for coronary heart disease risk stratification in chronic kidney disease. JACC Cardiovasc Imaging. (2021) 14(3):669–82. 10.1016/j.jcmg.2020.05.035 32828780

[17] Branny M. CT Coronary angiography and its role in diagnosing coronary disease. Interv Akut Kardiol. (2011) 10(SupplD):11–4.

[18] Wang LW Fahim MA Hayen A Mitchell RL Lord SW Baines LA Cardiac testing for coronary artery disease in potential kidney transplant recipients: a systematic review of test accuracy studies. Am J Kidney Dis. (2011) 57(3):476–87. 10.1053/j.ajkd.2010.11.018 21257239

[19] Ko DT Tu JV Austin PC Wijeysundera HC Samasashvili Z Guo H Prevalence and extent of obstructive coronary artery disease among patients undergoing elective coronary catheterization in New York state and Ontario. JAMA. (2013) 310(2):163–9. 10.1001/jama.2013.7834 23839750

[20] König J Möckel M Mueller E Bocksch W Baid-Agrawal S Babel N Risk-stratified cardiovascular screening including angiographic and procedural outcomes of percutaneous coronary interventions in renal transplant candidates. J Transplant. (2014) 2014:854397. 10.1155/2014/854397 25045528 PMC4089839

[21] Baman JR Knapper J Raval Z Harinstein ME Friedewald JJ Maganti K Preoperative noncoronary cardiovascular assessment and management of kidney transplant candidates. Clin J Am Soc Nephrol. (2019) 14:1670–6. 10.2215/CJN.03640319 31554619 PMC6832054

[22] Tabriziani H Baron P Abudayyeh I Lipkowitz M. Cardiac risk assessment for end-stage renal disease patients on the renal transplant waiting list. Clin Kidney J. (2019) 12(4):576–85. 10.1093/ckj/sfz039 31384451 PMC6671484

[23] Hart A Weir MR Kasiske BL. Cardiovascular assessment in kidney transplantation. Kidney Int. (2015) 87(3):527–34. 10.1038/ki.2014.335 25296093

[24] Gill JS. Screening transplant waitlist candidates for coronary artery disease. Clin J Am Soc Nephrol. (2019) 14:112–4. 10.2215/CJN.10510918 30593488 PMC6364538

[25] De Lima JJ Sabbaga E Vieira MLC de Paula FJ Ianhez LE Krieger EM Coronary angiography is the best predictor of events in renal transplant candidates compared with noninvasive testing. Hypertension. (2003) 43:263–8. 10.1161/01.HYP.0000087889.60760.87 12913060

[26] Katta N Balla S Velagapudi P Mittal M Agrawal H Kumar A Preoperative cardiac evaluation in kidney transplant patients: is coronary angiography superior? A focused review. Adv Perit Dial. (2016) 32:32–8. 28988587

[27] Charytan D Kuntz RE Mauri L DeFilippi C. Distribution of coronary artery disease and relation to mortality in asymptomatic hemodialysis patients. Am J Kidney Dis. (2007) 49:409–16. 10.1053/j.ajkd.2006.11.042 17336702

[28] Palepu S Prasad R. Screening for cardiovascular disease before kidney transplantation. World J Transplant. (2015) 5(4):276–86. 10.5500/wjt.v5.i4.276 26722655 PMC4689938

[29] Bangalore S Maron DJ O’Brien SM Fleg JL Kretov EI Briguori C Management of coronary disease in patients with advanced kidney disease. N Engl J Med. (2020) 382:1608–18. 10.1056/NEJMoa1915925 32227756 PMC7274537

[30] De Lima JJG Gowdak LHW de Paula FJ Ianhez LE Ramires JAF Krieger EM. Validation of a strategy to diagnose coronary artery disease and predict cardiac events in high-risk renal transplant candidates. Coron Artery Dis. (2010) 21:164–7. 10.1097/MCA.0b013e328332ee5e 20299981

[31] Kumar N Baker CSR Chan K Duncan N Malik I Frankel A Cardiac survival after pre-emptive coronary angiography in transplant patients and those awaiting transplantation. Clin J Am Soc Nephrol. (2011) 6(8):1912–9. 10.2215/CJN.08680910 21737845 PMC3359544

[32] Kahn MR Fallahi A Kim MC Robbins MJ. Coronary artery disease in a large renal transplant population: implications for management. Am J Transplant. (2011) 11(12):2665–74. 10.1111/j.1600-6143.2011.03734.x 21920018

[33] Nevis IF Matthew A Novick RJ Parikh CR Devereaux PJ Natarajan MK Optimal method of coronary revascularization in patients receiving dialysis: systematic review. Clin J Am Soc Nephrol. (2009) 4:369–78. 10.2215/CJN.02640608 19218473 PMC2637598

[34] Tavakol M Ashraf S Brener SJ. Risks and complications of coronary angiography: a comprehensive review. Glob J Health Sci. (2012) 4(1):65–93. 10.5539/gjhs.v4n1p65 22980117 PMC4777042

[35] Janousek R Krajina A Peregrin JH Dusilova-Sulkova S Renc O Hajek J Effect of intravascular iodinated contrast media on natural course of end-stage renal disease progression in hemodialysis patients: a prospective study. Cardiovasc Intervent Radiol. (2010) 33(1):61–6. 10.1007/s00270-009-9715-3 19830486

[36] Moranne O Willoteaux S Pagniez D Dequiedt P Boulanger E. Effect of iodinated contrast agents on residual renal function in PD patients. Nephrol Dial Transplant. (2006) 21(4):1040–5. 10.1093/ndt/gfi327 16352623

[37] Oloko A Taireja H Davis A McCormick B Clark E Akbari A Does iodinated contrast affect residual renal function in dialysis patients? a systematic review and meta-analysis. Nephron. (2020) 144(4):176–84. 10.1159/000505576 32155642

[38] Nielsen YW Thomsen HS. Current evidence of contrast medium-induced nephropathy (CIN) after administration of low-osmolarity iodine-based contrast agents. Curr Radiol Rep. (2017) 5:52. 10.1007/s40134-017-0244-6

[39] Chang TI Shilane D Kazi DS Montez-Rath ME Hlatky MA Winkelmayer WC. Multivessel coronary artery bypass grafting versus percutaneous coronary intervention in ESRD. J Am Soc Nephrol. (2012) 23(12):2042–9. 10.1681/ASN.2012060554 23204445 PMC3507369

[40] Chan W Ivanov J Ko D Fremes S Rao V Jolly S Clinical outcomes of treatment by percutaneous coronary intervention versus coronary artery bypass graft surgery in patients with chronic kidney disease undergoing index revascularization in Ontario. Circ Cardiovasc Interv*.* (2015) 8:8. 10.1161/CIRCINTERVENTIONS.114.001973 25582144

[41] Hemmelgarn BR Southern D Culleton BF Mitchell LB Knudtson ML Ghali WA. Survival after coronary revascularization among patients with kidney disease. Circulation. (2004) 110(14):1890–5. 10.1161/01.CIR.0000143629.55725.D9 15451786

[42] Shroff GR Herzog CA. Coronary revascularization in patients with CKD stage 5D: pragmatic considerations. J Am Soc Nephrol. (2016) 27:3521–9. 10.1681/ASN.2016030345 27493258 PMC5118494

[43] Bangalore S Guo Y Samadashvili Z Blecker S Xu J Hannan EL. Everolimus-eluting stents or bypass surgery for multivessel coronary disease. N Eng J Med. (2015) 372:1213–22. 10.1056/NEJMoa1412168 25775087

[44] Zoccali C Benedetto F Mallamaci F Tripepi G Giacone G Cataliotti A Prognostic value of echocardiographic indicators of left ventricular systolic function in asymptomatic dialysis patients. J Am Soc Nephrol. (2004) 15:1029–37. 10.1097/01.asn.0000117977.14912.91 15034106

[45] Wang AY Wang M Lam CW Chan HIS Zhang Y Sanderson JE. Left ventricular filling pressure by Doppler echocardiography in patients with end-stage renal disease. Hypertension. (2008) 52:107–14. 10.1161/HYPERTENSIONAHA.108.112334 18474835

[46] Lenihan CR Montez-Rath ME Scandling JD Turakhia P Winkelmayer WC. Outcomes after kidney transplantation of patients previously diagnosed with atrial fibrillation. Am J Transplant. (2013) 62(5):877–9. 10.1111/ajt.12197 PMC3670777 23721555

[47] Jeloka TK Ross H Smith R Huang M Fenton S Cattran D Renal transplant outcome in high cardiovascular risk recipients. Clin Transplant. (2007) 21:609–14. 10.1111/j.1399-0012.2007.00695.x 17845634

[48] Albakr RB Bargman JM. A comparison of hemodialysis and peritoneal dialysis in patients with cardiovascular disease. Cardiol Clin. (2021) 39(3):447–53. 10.1016/j.ccl.2021.04.013 34247757

[49] Banshodani M Kawanishi H Moriishi M Shintaku S Tsuchiya S. Association between dialysis modality and cardiovascular diseases: a comparison between peritoneal dialysis and hemodialysis. Blood Purif. (2020) 49(3):302–9. 10.1159/000504040 31851981

[50] Refaat H Sany D Mohab A Ezzat H. Comparing dialysis modality and cardiovascular mortality in patients on hemodialysis and peritoneal dialysis. Adv Perit Dial. (2016) 32:22–31. 28988586

[51] Trespalacios FC Taylor AJ Agodoa LY Bakris GL Abott KC. Heart failure as a cause for hospitalization in chronic dialysis patients. Am J Kidney Dis. (2003) 41(6):1267–77. 10.1016/s0272-6386(03)00359-7 12776280

[52] Satyan S Rocher LL. Impact of kidney transplantation on the progression of cardiovascular disease. Adv Chronic Kidney Dis. (2004) 11(3):274–93. 10.1053/j.arrt.2004.04.010 15241742

[53] Lentine KL Xiao HX Brennan DC Schnitzler MA Villainess TC Abbott KC The impact of kidney transplantation on heart failure risk varies with candidate body mass index. Am Heart J. (2009) 158(6):972–82. 10.1016/j.ahj.2009.10.009 19958864 PMC2804249

[54] Wali RK Wang GS Gottlieb SS Bellumkonda L Hansalia R Ramos E Effect of kidney transplantation on left ventricular systolic dysfunction and congestive heart failure in patients with end-stage renal disease. J Am Coll Cardiol. (2005) 45(7):1051–60. 10.1016/j.jacc.2004.11.061 15808763

[55] Edmonston DL Parikh KS Rajagopal S Shaw LK Abraham D Grabner A Pulmonary hypertension subtypes and mortality in CKD. Am J Kidney Dis. (2020) 75(5):713–24. 10.1053/j.ajkd.2019.08.027 31732231 PMC7183902

[56] Issa N Krowka MJ Griffin MD Hickson LJ Stegall MD Cosio F. Pulmonary hypertension is associated with reduced patient survival after kidney transplantation. Transplantation. (2008) 86(10):1384–8. 10.1097/TP.0b013e318188d640 19034007

[57] Naranjo M Lo KB Mezue K Rangaswami J. Effects of pulmonary hypertension and right ventricular function in short and long-term kidney function. Current Cardiol Rev. (2019) 15(1):3–11. 10.2174/1573403X14666181008154215 PMC6367698 30306876

[58] Lentine KL Villines TC Axelrod D Kaviratne S Weir MR Costa SP. Evaluation and management of pulmonary hypertension in KTR: concepts and controversies. Transplantation. (2017) 101(1):166–81. 10.1097/TP.0000000000001043 26985742

[59] Ewing EC Edwards AR. Cardiovascular disease assessment prior to kidney transplantation. Methodist DeBakey Cardiovasc J. (2022) 18(4):50–61. 10.14797/mdcvj.1117 36132581 PMC9461695

[60] Siddiqui MU Junarta J Marhefka GD. Coronary revascularization versus optimal medical therapy in renal transplant candidates with coronary artery disease: a systematic review and meta-analysis. J Am Heart Assoc. (2022) 11(4):e023548. 10.1161/JAHA.121.023548 35132876 PMC9245820

[61] Roberts CA Stern DL Radio SJ. Asymmetric cardiac hypertrophy at autopsy in patients who received FK506 (tacrolimus) or cyclosporine A after liver transplant. Transplantation. (2002) 74(6):817–21. 10.1097/00007890-200209270-00015 12364862

[62] Choudhary R Sastry BKS. Subramanyam. Int J Cardiol. (2005) 105(3):327–31. 10.1016/j.ijcard.2005.04.006 16274778

[63] Elezaby A Dexheimer R Sallam K. Cardiovascular effects of immunosuppression agents. Front Cardiovasc Med. (2022) 9:981838. 10.3389/fcvm.2022.981838 36211586 PMC9534182

[64] KDIGO Clinical practice guideline on the evaluation and management of candidates for kidney transplantation. Transplantation. (2020) 104:4S. 10.1097/TP.0000000000003136 32301874

